# Treatment Strategy for Post-Obstetric Rectovaginal Fistula without Requiring Diverting Stoma: A Retrospective Analysis of 46 Cases Demonstrating the Superiority of Our Institution’s Original Technique

**DOI:** 10.31662/jmaj.2025-0474

**Published:** 2026-07-17

**Authors:** Koichiro Murakami, Yumi Nishimura, Tomoyuki Mizukuro

**Affiliations:** 1 Department of Surgery, Soshinkai Nagaokakyo Hospital, Kyoto, Japan; 2 Department of Digestive Surgery, Shiga University of Medical Science, Otsu, Japan

**Keywords:** obstetric trauma, rectovaginal fistula, perineal body reconstruction, diverting stoma, fistula closure

## Abstract

**Background:**

Post-obstetric rectovaginal fistula significantly impairs women’s quality of life, and the indication for diverting stoma creation remains controversial. Despite numerous international reports indicating that diverting the stoma is unnecessary for post-obstetric rectovaginal fistula, many institutions continue to perform stoma creation in clinical practice.

**Methods:**

We retrospectively analyzed 46 cases of post-obstetric rectovaginal fistula treated with perineal body reconstruction over 3 years. Patient demographics, fistula characteristics, treatment history at other institutions, surgical outcomes, and postoperative course were evaluated. The primary outcome was the absence of fistula recurrence during a 12-month follow-up period.

**Results:**

The median age was 34 years, the median body mass index was 21, the median fistula size was 2 mm, and the median duration from onset to surgery was 268 days. The majority of cases involved severe obstetric trauma with grade 4 perineal lacerations. Seven patients (15.2%) were recommended stoma creation at other institutions, and a total of 15 surgeries were performed at other institutions. Stable surgical outcomes were achieved with a median operative time of 130 minutes and a median blood loss of 250 mL. During the 12-month follow-up period, favorable results were obtained in 44 cases (95.7%), with one missed concomitant high fistula subsequently treated successfully via a transvaginal approach, and one suspected recurrence reported by telephone at 24 months (after completion of follow-up) without clinical confirmation.

**Conclusions:**

Our institution’s original perineal body reconstruction technique achieved a high cure rate without requiring a diverting stoma creation. Typical post-obstetric rectovaginal fistulas develop at the lowest point where rectal pressure is highest, making perineal body reconstruction effective for the majority of cases. However, in patients with extensive perineal laceration scarring on digital rectal examination, preoperative fistulography should be considered to rule out concomitant high fistulas. Diverting stoma creation for post-obstetric rectovaginal fistula is generally unnecessary.

## Introduction

Rectovaginal fistula significantly impairs women’s quality of life. Obstetric trauma, particularly sphincter injury following grade 3-4 perineal lacerations, is a major etiology because the close proximity of the vagina and rectum can lead to fistula formation. The indication for diverting stoma creation in treating this condition has been debated for many years.

International literature predominantly reports that diverting stoma creation is unnecessary for post-obstetric rectovaginal fistula. Pinto et al. reported achieving a 95% cure rate without requiring stoma creation in 113 cases. Similarly, Tsang et al. questioned the necessity of stoma creation and concluded that treatment is possible with appropriate surgical techniques without requiring a stoma.

However, in clinical practice in Japan, diverting stoma creation is proposed and performed for post-obstetric rectovaginal fistula at university hospitals and many high-volume centers. This indicates a significant discrepancy between literature-based evidence and actual clinical practice.

Our institution has established an original treatment strategy using perineal body reconstruction without requiring a diverting stoma for post-obstetric rectovaginal fistula, achieving favorable treatment outcomes. This study validates the efficacy of our treatment strategy through retrospective analysis of 46 cases and reconsiders the necessity of stoma creation.

## Materials and Methods

### Study population

We included 46 cases of post-obstetric rectovaginal fistula that underwent surgery at our institution between July 2022 and June 2025. All cases involved rectovaginal fistula resulting from perineal lacerations during delivery. Fistulas due to malignancy, inflammatory bowel disease, or radiation were excluded.

### Study design

We conducted a retrospective analysis using medical records. The evaluation items included patient demographics (age, delivery mode, perineal laceration grade, and duration from onset to surgery), fistula characteristics (fistula size), treatment history at other institutions (recommendation for stoma creation, actual stoma creation, and number of surgeries for rectovaginal fistula), and intraoperative variables (operative time, blood loss, and number of perineal body sutures).

### Outcome measures

The primary outcome was the absence of fistula recurrence during the 12-month follow-up period. The 12-month follow-up was set as the standard observation period for all patients who completed follow-up. Secondary outcomes included postoperative complications (including wound infection, fecal incontinence, and chronic pain), changes in fecal incontinence severity (assessed by Wexner score), and improvement in sexual function.

### Surgical technique

All procedures were performed with patients in the jackknife (prone) position under saddle block anesthesia. Our institution’s original perineal body reconstruction was performed in all cases following these steps:

1. Transverse perineal incision and subcutaneous dissection (Figure 1)

2. Adequate dissection of rectal and vaginal walls, confirmation of fistula presence (Figure 2)

3. Excision of fistulous tract, including vaginal and rectal walls, when a fistula is confirmed (Figure 3)

4. Reliable closure of the rectal wall with vertical mattress sutures and repair of the vaginal wall with continuous sutures

5. Confirmation that bilateral perineal bodies are adequately dissected with good mobility, allowing midline crossing (Figure 4)

6. Three-dimensional repair of the perineal body using 3-0 absorbable sutures (Figure 5)

7. Vertical closure of the perineal area (Figure 6)

**Figure 1.**
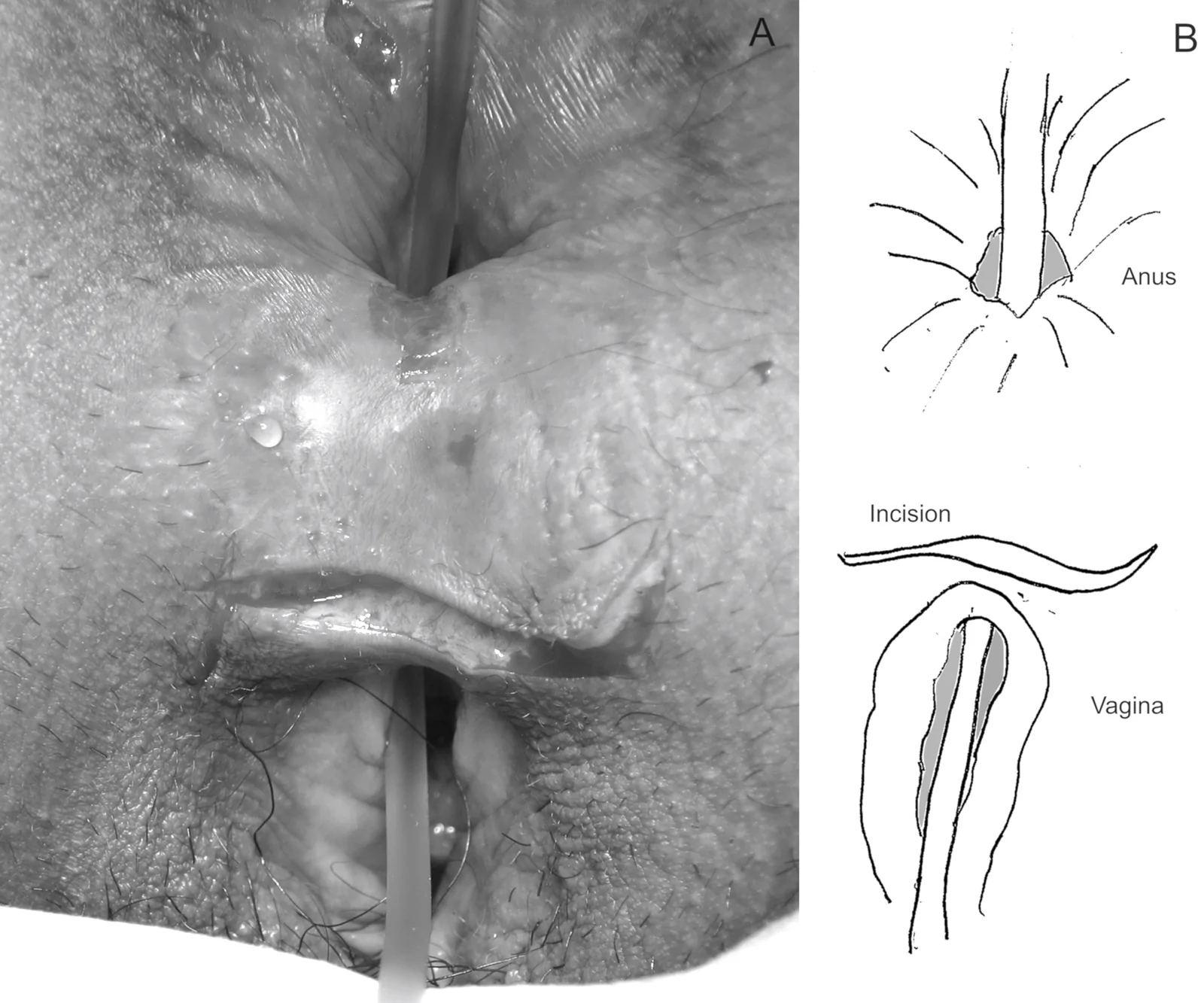
Initial surgical exposure and skin incision. (A) Intraoperative photograph showing the initial surgical field immediately after skin incision for rectovaginal fistula repair. The anus is positioned superiorly and the vagina inferiorly in the image. An 8-French Nelaton catheter is inserted through the fistulous tract to serve as a landmark. Note the shortened distance between the anal and vaginal openings due to a perineal body defect. A transverse S-shaped skin incision is placed on the vaginal side to avoid injury to the residual anal sphincter muscle. (B) Schematic diagram illustrating the surgical approach, with the rectum and vagina clearly differentiated.

**Figure 2.**
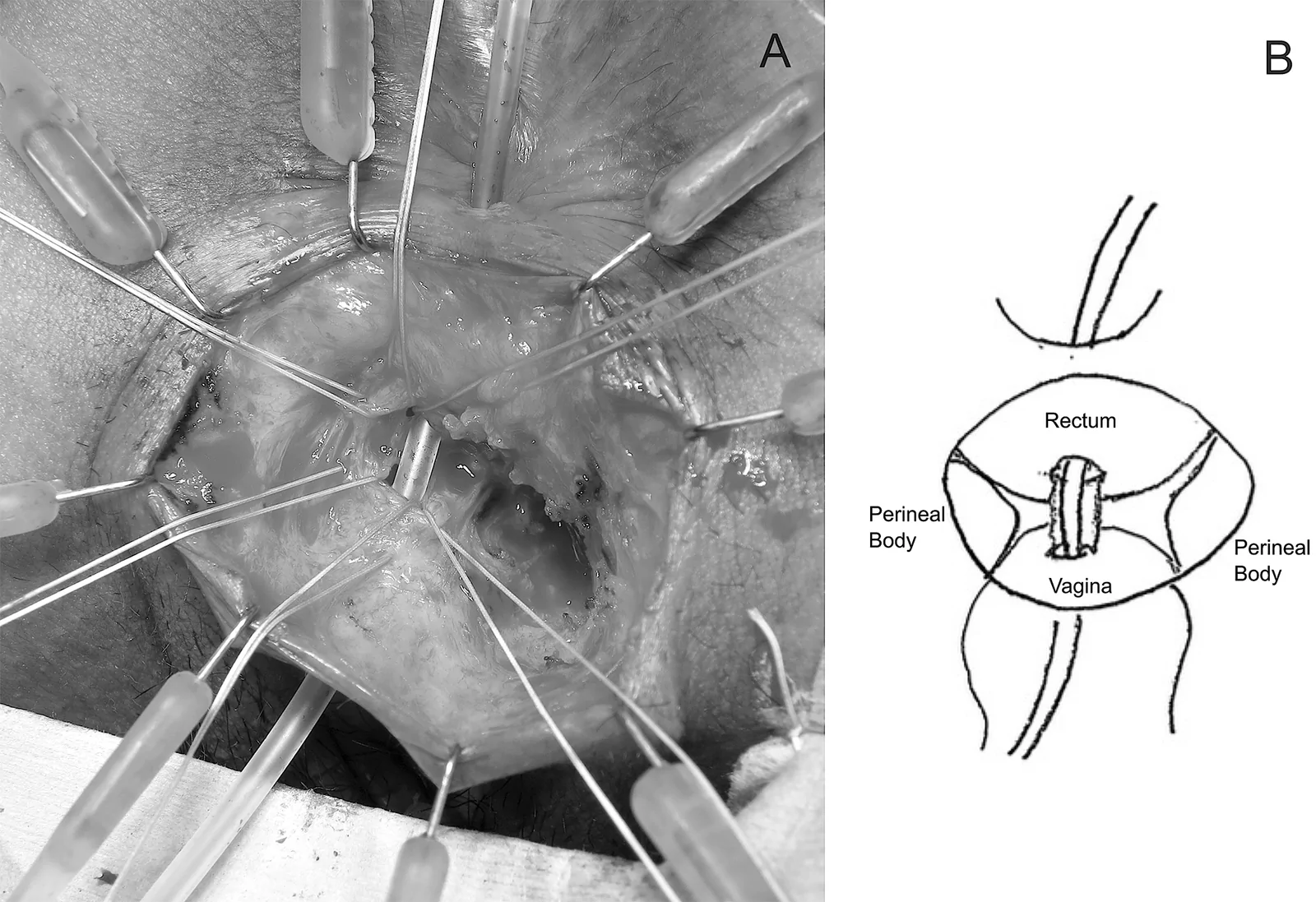
Fistulous tract exposure and luminal assessment. (A) Surgical field showing advancement of dissection to reach the fistulous tract, which has been opened to visualize the internal lumen. Dissection proceeds toward both vaginal and rectal sides to ensure complete removal of all fistulous tissue without residual tract remnants. (B) Schematic representation of the fistulous tract exposure and the extent of dissection required for complete tract removal.

**Figure 3.**
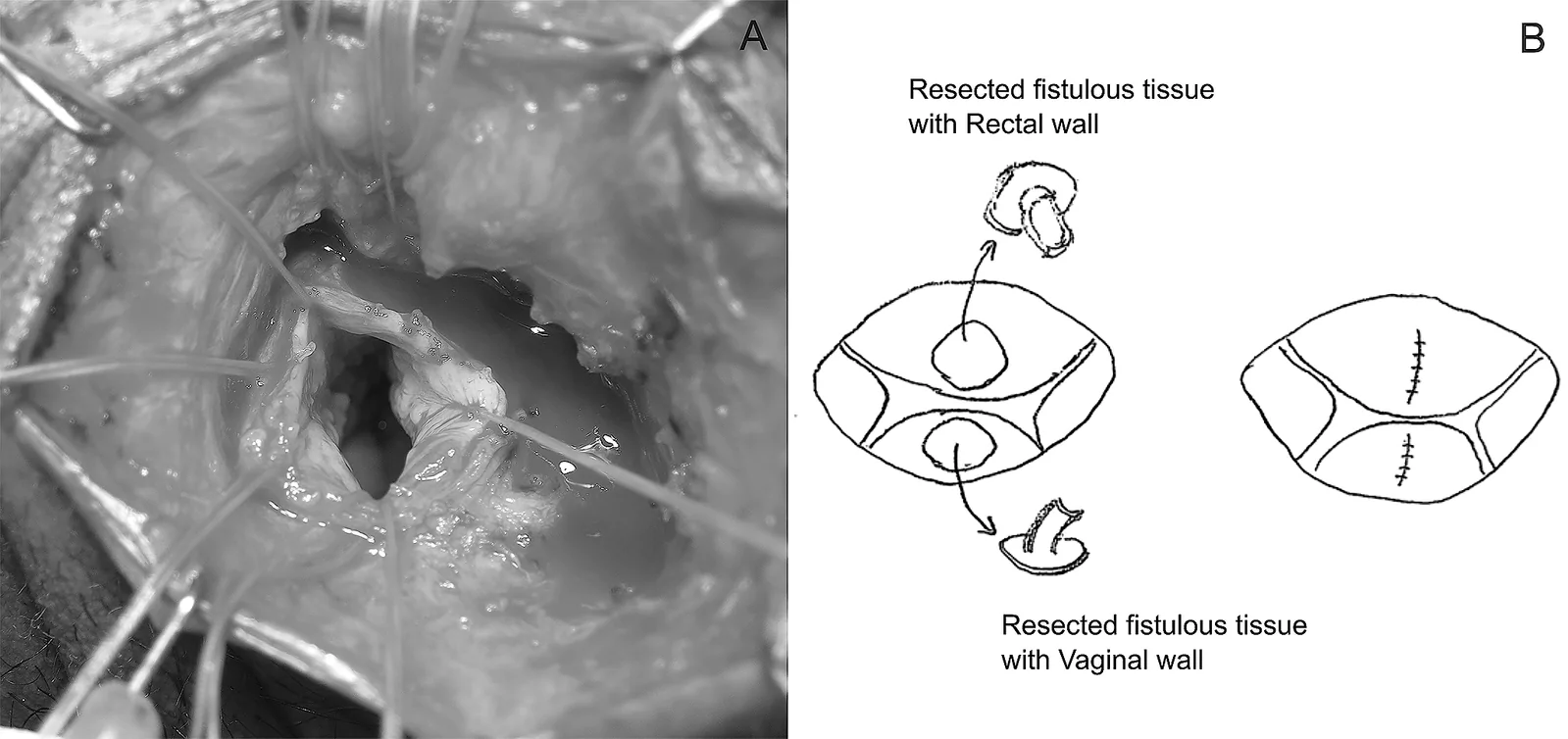
Fistulous tract excision and debridement of contaminated tissue. (A) Intraoperative view showing extension of fistulous tract excision to the vaginal side. The contaminated vaginal wall surrounding the fistular origin is excised en bloc with the tract. A similar en bloc excision of the contaminated rectal wall is performed on the rectal side. Even fistulas with diameters less than 1 cm may result in defects reaching approximately 5 cm after complete debridement of contaminated tissue. (B) Diagram illustrating the en bloc excision of the fistulous tract with portions of both rectal and vaginal walls, followed by separate suture closure of each luminal defect.

**Figure 4.**
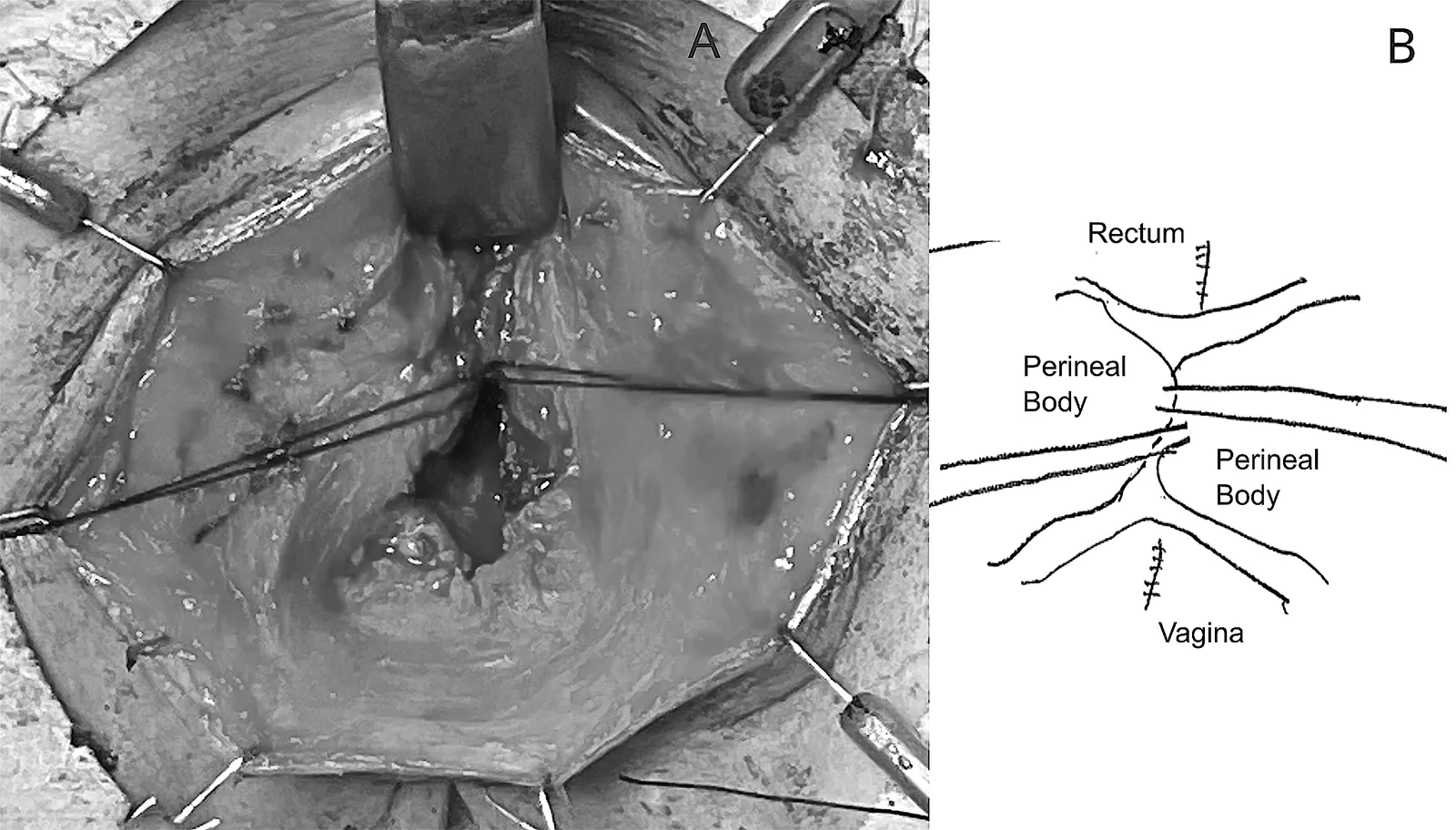
Perineal body assessment and tissue viability testing. (A) Intraoperative demonstration of traction suture placement on bilateral perineal body remnants. The sutures are crossed at the midline to assess tissue integrity and mobility. Adequate perineal body tissue should demonstrate elastic properties allowing mobilization across the midline without incorporating overlying rectal or vaginal walls. (B) Schematic illustration of the traction test to evaluate perineal body viability and elasticity.

**Figure 5.**
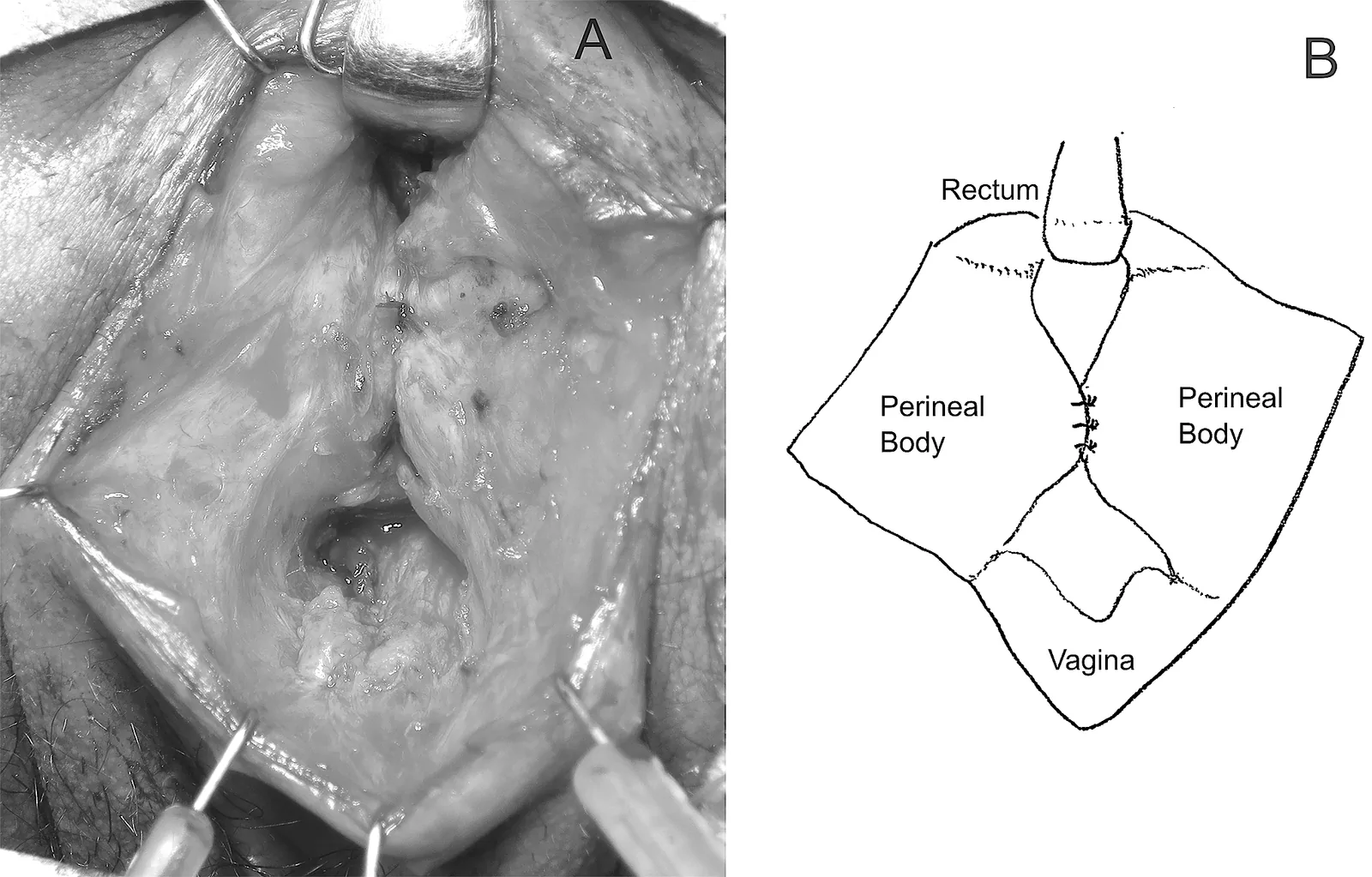
Perineal body reconstruction with multilayer suturing. (A) Intraoperative photograph showing multilayer suture repair of the perineal body. Reconstruction is performed in 4-5 distinct layers from the deepest to most superficial planes, using 3-0 absorbable sutures with typically more than 10 interrupted sutures in total across all layers to achieve three-dimensional anatomical restoration of the perineal body. (B) Schematic diagram illustrating the layered reconstruction technique for optimal perineal body restoration.

**Figure 6.**
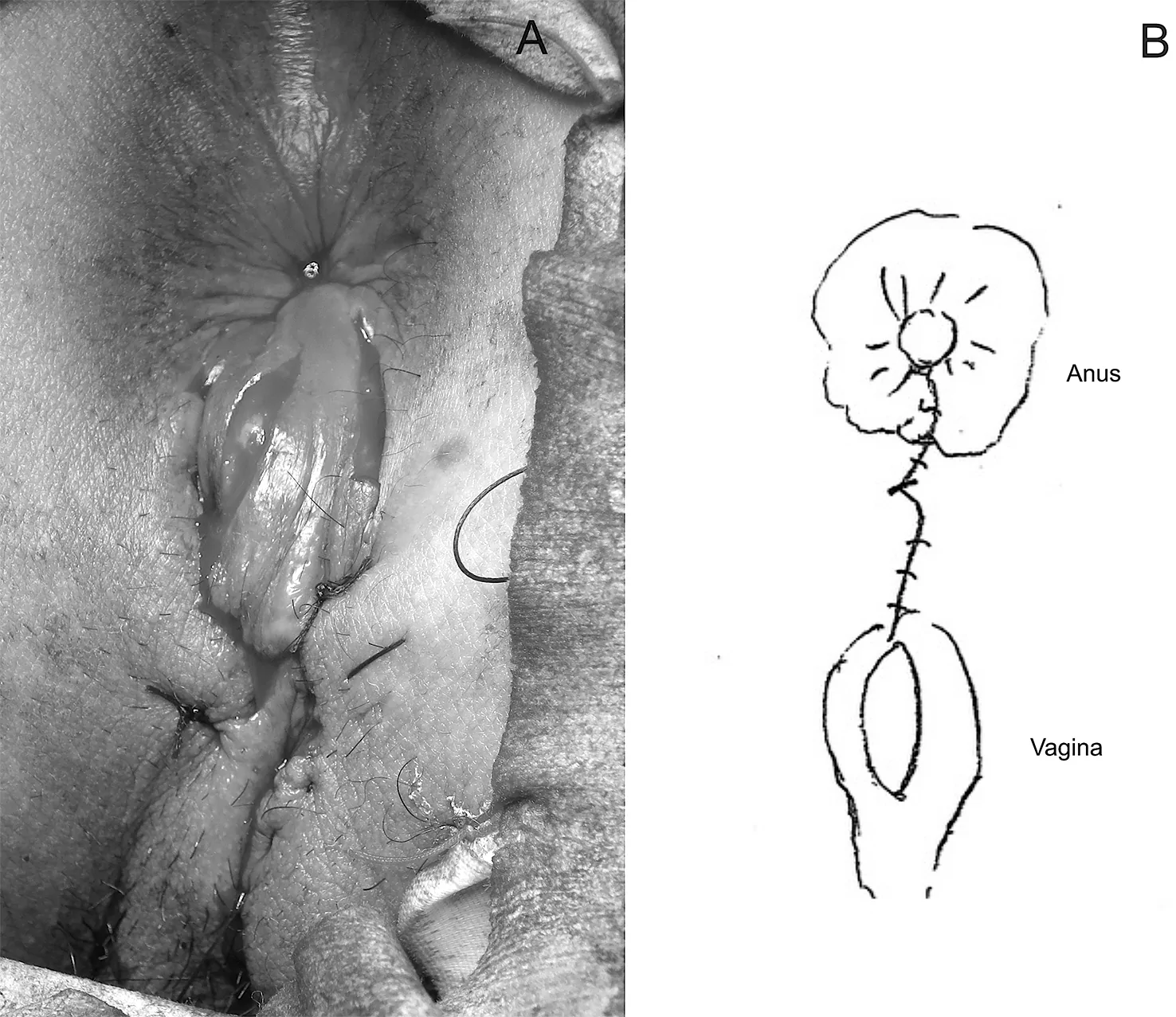
Final surgical result and wound closure. (A) Completed surgical repair showing subcutaneous interrupted sutures and continuous skin closure with perineal reconstruction. Anal swelling frequently occurs postoperatively, necessitating skin-only relaxing incisions to prevent sphincter muscle injury. The successful perineal body reconstruction results in increased distance between vagina and rectum (restored perineal length), with wound closure performed in a vertical direction. (B) Schematic diagram showing the final anatomical result with restored perineal anatomy and proper anal-vaginal separation.

Primary fistula closure was performed in all cases without requiring stoma creation. No drains were placed.

### Statistical analysis

Continuous variables are expressed as median (range), and categorical variables as number of cases (%). All statistical analyses were performed using R version 4.4.2 (R Foundation for Statistical Computing, Vienna, Austria).

### Ethics statement

This study was approved by the institutional review board of Tesseikai Neurosurgical Hospital (approval number: 2025-88) and was conducted in accordance with the ethical standards of the Declaration of Helsinki. Informed consent was obtained from all participants.

## Results

### Patient demographics

Patient demographics for the 46 cases are shown in Table 1. The median age was 34 years (26-62 years). Grade 4 perineal laceration was most common with 18 cases (39.1%), followed by no recorded grade in 17 cases (37.0%), grade 2 in 7 cases (15.2%), and grade 3 in 3 cases (6.5%). The median duration from onset to surgery was 268 days.

**Table 1.** Patient Demographics and Clinical Characteristics (n = 46).

Parameter	Median (range) or number (%)
Age (years)	34 (26-62)
Fistula size (mm)	2 (0-20)
Duration from onset to surgery (days)	269 (45-6,271)
Grade 4 perineal laceration	18 (39.1)
Grade 3 perineal laceration	3 (6.5)
Grade 2 perineal laceration	7 (15.2)
Perineal laceration grade unknown	17 (37.0)
Previous surgery at other institutions	15 surgeries in 11 patients (23.9%)
Stoma recommendation from other institutions	7 patients (15.2%)

### Fistula characteristics

The median fistula size was 2 mm (0-20 mm). While relatively small fistulas were common, cases with large fistulas were also included.

### Surgical outcomes

The median operative time was 130 minutes (84-198 minutes), the median blood loss was 250 mL (55-991 mL), and the median number of sutures was 14 (8-18) (Table 2). No serious intraoperative complications occurred.

**Table 2.** Surgical Outcomes and Postoperative Results (n = 46).

Parameter	Median (range) or number (%)
Operative time (minutes)	130 (84-198)
Blood loss (mL)	250 (55-991)
Number of sutures	14 (8-18)
Postoperative complications	10 (21.7%)
Recurrence	2 (4.3%)
Follow-up period (months)	12 (6-12)

### Treatment results

Postoperative complications occurred in 10 cases (21.7%). Complications included fecal incontinence in five cases, missed concomitant high fistula in one case, chronic pain in one case, and subcutaneous infection in two cases. Subcutaneous infections were treated with Seton drainage in one case and conservative antibiotic therapy in another. Regarding fecal incontinence, all affected patients had severe preoperative incontinence (Wexner score ≥12) that improved postoperatively.

During the 12-month follow-up period, favorable results were obtained in 44 cases (95.7%) with perineal body reconstruction alone. Among the two cases that did not achieve the primary outcome, one had a missed concomitant high fistula approximately 10 cm proximal to the anus (identified on postoperative fistulography), which was subsequently treated successfully via a transvaginal approach at 18 months. The other reported suspected recurrence by telephone at 24 months (after completion of the standard follow-up period) without clinical confirmation, as the patient was unable to visit due to residing in a distant area.

Sexual function improved in all cases where assessment was possible, with two patients achieving postoperative pregnancy and vaginal delivery, and three patients delivering via cesarean section.

## Discussion

### Summary of key findings

This study demonstrates that our institution’s original perineal body reconstruction technique achieved a 95.7% success rate (44/46 cases) in treating post-obstetric rectovaginal fistula without requiring a diverting stoma during the 12-month follow-up period. The technique proved effective even in challenging cases, including those in which stoma creation was recommended at other institutions (7 cases) and those with multiple prior surgical failures (11 patients with 15 prior surgeries).

### Comparison with previous literature

International literature is predominantly negative regarding the necessity of stoma creation for post-obstetric rectovaginal fistula. Pinto et al. reported a 95% cure rate without a stoma in 113 cases. Tsang and Rothenberger concluded that stoma creation is not routinely necessary. Hull et al. reported an 85% success rate in the non-stoma group, with no significant difference compared to the stoma group. Our results (95.7% success rate) are consistent with these reports.

### Comparison with other surgical techniques

Several surgical techniques have been described for rectovaginal fistula repair. The endorectal advancement flap (ERAF), first described by Rothenberger in 1982, has reported success rates of 65%-82%. While ERAF provides mucosal coverage, it does not address the underlying perineal body defect. Muscle flap repairs, such as the gracilis muscle flap, have reported success rates of approximately 72% but carry significant donor site morbidity. The transvaginal approach, as demonstrated in one of our cases, can be effective for high fistulas that are not accessible via the perineal route.

Our perineal body reconstruction technique offers several advantages: (1) it addresses both the fistula and the underlying anatomical defect; (2) it simultaneously improves sphincter function; (3) it restores normal perineal anatomy; and (4) it avoids the morbidity associated with stoma creation or muscle flap harvest.

### Pathophysiology and patient selection

Based on our experience, we propose the following rationale for patient selection and surgical technique. Typical post-obstetric rectovaginal fistulas develop at the lowest point of the rectovaginal septum, where rectal intraluminal pressure is highest during defecation and straining. At this location, the fistula is in close proximity to the disrupted perineal body, making perineal body reconstruction with robust tissue interposition an essential approach for the majority of post-obstetric fistulas. Conversely, for fistulas extending higher than the upper margin of the perineal body (approximately 3 cm from the anus), the rectal intraluminal pressure is significantly lower, and our experience suggests that fistula tract excision with careful suturing of the rectal and vaginal walls alone—without tissue interposition—is sufficient to achieve healing.

However, in rare cases with extensive perineal lacerations, concomitant high fistulas may develop in addition to the typical low fistula. Because most post-obstetric rectovaginal fistulas are located just above the dentate line and are typically not visualized on contrast studies, we had not routinely performed preoperative fistulography. The missed high fistula case taught us that this approach requires modification in selected patients. In that case, fistula tract excision and careful suturing of the rectal and vaginal walls without tissue interposition achieved complete healing at 18 months.

We now recommend that when digital rectal examination reveals extensive perineal laceration scarring, preoperative fistulography or other imaging studies (such as endoanal ultrasound or magnetic resonance imaging) should be actively performed to rule out concomitant high fistulas before proceeding with perineal body reconstruction alone. Patients with identified high fistulas should be counseled that alternative or additional surgical approaches may be necessary.

## Limitations

This study has several limitations. First, this is a single-center retrospective study without a control group. Second, one suspected recurrence was reported after completion of the 12-month follow-up period and could not be clinically confirmed due to the patient residing in a distant area. Although we could not confirm the actual cause, we hypothesize that a delayed local infection, such as a suture abscess, may have occurred beyond the follow-up period; if undrained, this could have led to perineal body dehiscence. Third, one case of missed high fistula highlights the importance of preoperative imaging in selected patients. Fourth, selection bias may exist. Fifth, the possibility of delayed local infections, such as suture abscess occurring beyond the standard 12-month follow-up period, suggests that extended follow-up of 18 or 24 months should be considered for patients who are able to attend regular follow-up visits. Future prospective comparative trials through multicenter collaboration are needed.

### Conclusions

Our institution’s original perineal body reconstruction technique achieved a high cure rate of 95.7% in 46 cases of post-obstetric rectovaginal fistula without requiring a diverting stoma during the 12-month follow-up period. Typical post-obstetric rectovaginal fistulas develop at the lowest point where rectal pressure is highest, and perineal body reconstruction is effective for the majority of these low fistulas. However, in rare cases with extensive perineal lacerations, concomitant high fistulas may coexist. Therefore, when digital rectal examination reveals extensive perineal laceration scarring, preoperative fistulography or other imaging studies should be actively performed to rule out concomitant high fistulas. For high fistulas not accessible via the perineal approach, alternative techniques such as the transvaginal approach can achieve successful outcomes. Additionally, considering the possibility of delayed local infections beyond the standard follow-up period, extended follow-up of 18 or 24 months should be considered for patients who are able to attend regular visits.

Our study results, consistent with international literature, demonstrate that stoma creation for post-obstetric rectovaginal fistula is generally unnecessary. Radical treatment is possible with appropriate surgical techniques and careful patient selection, significantly contributing to improved patient quality of life.

## Article Information

### Acknowledgments

We thank all patients who participated in this study and the medical staff who contributed to their care.

### Author Contributions

Conceptualization, Methodology, Surgical procedures, Data curation, Writing - original draft, Writing - review & editing, Project administration: Koichiro Murakami.

Methodology, Data analysis, Writing - review & editing, Validation: Yumi Nishimura.

Methodology, Investigation, Data collection, Writing - review & editing, Supervision: Tomoyuki Mizukuro.

### Conflicts of Interest

None

### IRB Approval Code and Name of Institution

This study was approved by Tesseikai Neurosurgical Hospital Institutional Review Board (Approval No. 2025-88).

### Technical Notes

All procedures were performed under saddle block anesthesia in the jackknife position. Written informed consent for publication of surgical images was obtained from all patients. Patient privacy was maintained through appropriate anonymization of identifying features.
